# In Situ Growth of W_2_C/WS_2_ with Carbon-Nanotube Networks for Lithium-Ion Storage

**DOI:** 10.3390/nano12061003

**Published:** 2022-03-18

**Authors:** Thang Phan Nguyen, Il Tae Kim

**Affiliations:** Department of Chemical and Biological Engineering, Gachon University, Seongnam-si 13120, Korea; phanthang87@gmail.com

**Keywords:** *WS*_2_, *W*_2_ *C*, hydrothermal method, carbon nanotubes, lithium-ion batteries

## Abstract

The combination of *W*_2_*C* and *WS*_2_ has emerged as a promising anode material for lithium-ion batteries. *W*_2_*C* possesses high conductivity but the *W*_2_*C*/*WS*_2_-alloy nanoflowers show unstable performance because of the lack of contact with the leaves of the nanoflower. In this study, carbon nanotubes (CNTs) were employed as conductive networks for in situ growth of *W*_2_*C*/*WS*_2_ alloys. The analysis of X-ray diffraction patterns and scanning/transmission electron microscopy showed that the presence of CNTs affected the growth of the alloys, encouraging the formation of a stacking layer with a lattice spacing of ~7.2 Å. Therefore, this self-adjustment in the structure facilitated the insertion/desertion of lithium ions into the active materials. The bare *W*_2_*C*/*WS*_2_-alloy anode showed inferior performance, with a capacity retention of ~300 mAh g^−1^ after 100 cycles. In contrast, the WCNT01 anode delivered a highly stable capacity of ~650 mAh g^−1^ after 100 cycles. The calculation based on impedance spectra suggested that the presence of CNTs improved the lithium-ion diffusion coefficient to 50 times that of bare nanoflowers. These results suggest the effectiveness of small quantities of CNTs on the in situ growth of sulfides/carbide alloys: CNTs create networks for the insertion/desertion of lithium ions and improve the cyclic performance of metal-sulfide-based lithium-ion batteries.

## 1. Introduction

The rise of graphene, transition-metal chalcogenides (TMCs), transition-metal oxides, and layered-structure transition-metal carbides/nitrides (MXenes) shows the significance and potential of 2D-layered nanomaterials, which can be applied in various fields such as displays, energy storage, energy conversion, and electronic devices [1,2,3,4,5,6,7,8,9,10,11,12,13,14,15,16,17,18]. TMC materials possess high theoretical lithium-storage capacity (~670 mAh g^−1^ with MoS_2_ and 433 mAh g^−1^ with *WS*_2_). However, the practical showed that a high abnormal capacity was recorded, which can contribute by conversion reaction, the derived solid electrolyte interface (SEI)-layer formation, or the high lithiation process in the interfacial lithium-storage spaces [19,20,21]. For example, Feng et al. fabricated *WS*_2_ nanoflakes for lithium-ion batteries (LIBs), which delivered a high initial discharge capacity of ~ 1700 mAh g^−1^ at a current of 47.5 mA g^−1^ [22]. Liu et al. synthesized mesoporous *WS*_2_, showing a high initial discharge capacity of ~1300 mAh g^−1^ [23]. However, the TMCs anode material with the conversion reaction could be significantly degraded due to the dissolution of the sulfur into electrolyte, creating a gel-like polymeric layer [24]. Recently, the combination of TMCs with MXenes has received significant attention owing to the tunable bandgap of TMCs, active edge of chalcogenide atoms with high conductivity of MXenes, high stability, and active edge of metal atoms [25,26,27,28]. For example, Zhao et al. developed vertical MoS_2_/Mo_2_C nanosheets on carbon paper, which maximized the active sites of the active edges and resulted in high electrocatalytic performance in the hydrogen-evolution reaction [29]. Cheng et al. used guar gum as the carbon source for nanoflower MoS_2_/Mo_2_C as an efficient sustainable electrocatalyst for the production of hydrogen gas [30]. Faizan et al. fabricated Mo_2_C stacked with MoS_2_ nanosheets for lithium-storage applications. Li et al. [31] modified the surface of *WS*_2_/*W*_2_*C* materials with N and S, which improved their electrochemical catalytic properties [28]. Nguyen et al. controlled the growth of *W*_2_*C*/*WS*_2_ nanoflowers via a hydrothermal method for use as stable anode materials in lithium-ion batteries (LIBs) [32,33]. The carbide MXenes possess high conductivity and stability; however, they are not highly active materials by themselves [6,7,8,9,10]. Their lithium-storage capability is low, and thus they could only be employed as additive materials [34,35,36,37]. Meanwhile, carbon nanotubes (CNTs) possess high conductivity and light weight and are popular network materials for enhancing connectivity in electronic applications [38,39]. Therefore, CNTs and derived carbon materials have been widely used as skeleton or network of the active materials for LIBs. For example, Lu et al. used a CNT/MoS_2_ composite as a binder-free anode material showing high performance in LIBs [40]. Chen et al. developed a FeS_2_/CNT composite material with a neural-network-like structure, which delivered a superior rate and high cycling performance in sodium-ion batteries [41]. The use of TMCs, MXenes, and CNTs in a system could combine their advantage such as physical, chemical stability, high conductivity, and high capability for lithium-storage applications.

In this study, *W*_2_*C*/*WS*_2_-alloy nanoflowers were fabricated with a small quantity of CNTs as a connective network using a hydrothermal method. The presence of a CNT network is not only effective for the formation of alloy flowers but also improves the electrochemical performance of the as-prepared anode materials in lithium storage. The structural changes and stable performance of *W*_2_*C*/*WS*_2_ in a CNT network (WCNT) were investigated and discussed.

## 2. Materials and Methods

### 2.1. Chemical Materials

Thioacetamide (TAA, C_2_H_5_NS, 99%), WCl_6_ powder (99.9%), multiwalled CNTs (>90%), and polyvinylidene fluoride (PVDF, M_W_ ~ 534,000) were purchased from Sigma-Aldrich Inc. (St. Louis, MO, USA). Super-P amorphous carbon black (C, approximately 40 nm, 99.99%) and absolute ethanol were purchased from Alpha Aesar, Inc. (Ward Hill, MA, USA). All materials were used as received. WCl_6_ was stored in an Ar-filled glove box.

### 2.2. Synthesis of WCNT

The WCNT was prepared using a modified procedure for *W*_2_*C*/*WS*_2_ nanoflower synthesis [33]. CNTs were dispersed in ethanol using sonication. Then, 0.6 g WCl_6_ was added to 4 mL of the CNT solution with an adjusted weight ratio of CNT: WCl_6_ of 5, 10, and 15%. TAA (1.2 g) was dispersed in a separate vessel containing 4 mL of absolute ethanol. The TAA solution was then quickly mixed with the WCl_6_/CNT solution and stirred for 5 min. Then, 10 mL of deionized (DI) water was added to the solution, and the mixture was transfer into a 40 mL polypropylene-lined autoclave and heated at 250 °C for 12 h. The obtained powder was washed four times with ethanol and DI water and dried in a vacuum oven at 60 °C. The samples with different quantities of CNTs (5, 10, and 15%) were marked as WCNT01/02/03, respectively.

### 2.3. Material Characterization

The structures of *W*_2_*C*/*WS*_2_ and WCNT samples were determined using X-ray diffraction (XRD, D/MAX-2200 Rigaku, Tokyo, Japan) over the 2θ range of 10–70° and transmission electron microscopy (TEM, TECNAI G2F30, FEI Corp., Hilsboro, OR, USA). Their morphologies were analyzed using field emission scanning electron microscopy (FESEM, SIGMA HD, Carl Zeiss, Jena, Germany) at an accelerating voltage of 5 kV. Thermogravimetric analysis (TGA) was measured using a thermal analyzer (Q600 SDT, TA Instruments, New Castle, DE, USA).

### 2.4. Electrochemical Measurements

The *W*_2_*C*/*WS*_2_ and WCNT anode materials were evaluated by assembling half-cell LIBs using a coin-type cell (CR 2032, Rotech Inc., Gwangju, Korea) with a lithium reference electrode. The active materials were mixed with carbon super P and PVDF (weight ratio of 70:15:15) in a n-methyl-2-pyrrolidone solution to form a slurry, which was then coated on copper foil using a doctor blade. The working electrodes were dried in a vacuum oven at 70 °C for 24 h to remove the solvent. The anodes were punched into 12 mm circular disks. The loading mass of the active materials was ~1.0–1.3 mg. LIBs were assembled in an Ar-filled glove box using 1 M LiPF_6_ in ethylene carbonate/diethylene carbonate (EC:DEC = 1:1 by volume) as the electrolyte. Cyclic voltammetry (CV) tests and electrochemical impedance spectroscopy (EIS) were performed using a battery-cycle tester (WBCS3000, WonAtech, Seocho-gu, Seoul, Korea) over the voltage range of 0.01–3.0 V vs. *Li*/*Li*^+^ and frequency range from 100 kHz to 0.1 Hz, respectively. The cycling stabilities were measured over the voltage range of 0.01–3.00 V using a ZIVE MP1 (WonAtech, Seocho-gu, Seoul, Korea).

## 3. Results

**Figure 1:**
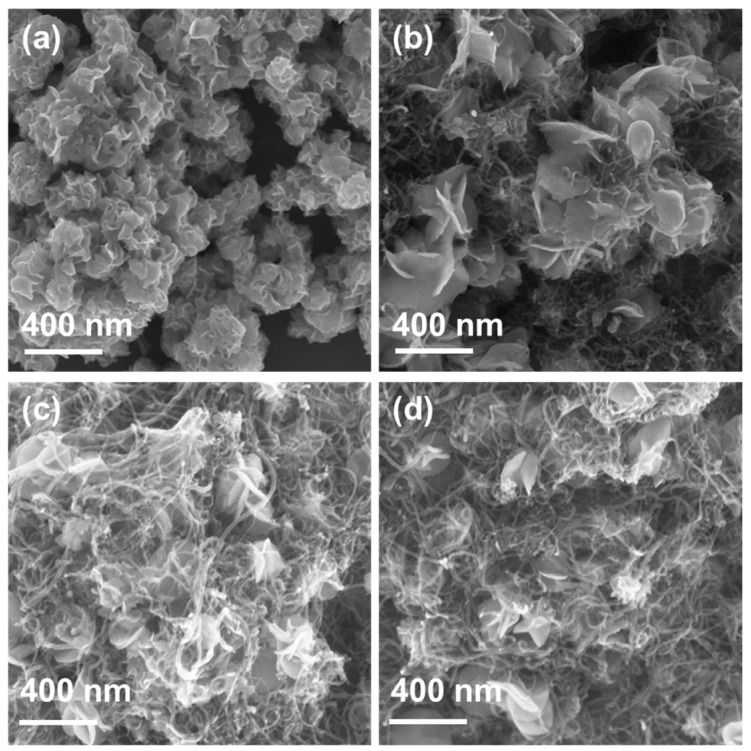
FESEM images of (**a**) *W*_2_*C*/*WS*_2_ (**b**) WCNT01, (**c**) WCNT02, and (**d**) WCNT03 samples.

The morphologies of the *W*_2_*C*/*WS*_2_ nanoflowers and the WCNT samples are shown in Figure 1. The sizes of nanoflowers range from 100 to 300 nm with many leaves, which consist of 2D nanosheets, as shown in Figure 1a. The presence of CNTs in the samples reduced the number of leaves. All the alloy nanoflowers were wrapped in the CNT network. In addition, at a low concentration of CNTs in the WCNT01 sample, the *W*_2_*C*/*WS*_2_ nanoflowers grew to a larger size of ~300–400 nm, as illustrated in Figure 1b. At above 10% of CNTs, the size of nanoflowers decreased to 200–300 nm, as shown in Figure 1c,d. At lower quantity of CNTs (2 and 3 wt%), the separate growth of *W*_2_*C*/*WS*_2_ nanoflowers was found (data not shown), indicating the nonuniformity. Therefore, the minimum content for the effective coverage of *W*_2_*C*/*WS*_2_ was 5 wt% CNTs. It is noteworthy that the presence of CNTs could act as a seed point for growth of *W*_2_*C*/*WS*_2_ nanoflowers. In the bare *W*_2_*C*/*WS*_2_ nanoflower, their leaves were bended around a center. In WCNT samples, the leaf surface was flat, resulting in an increase in the flower size. However, the increased quantity of CNTs could occupy more spaces in solution, which could limit the growth of *W*_2_*C*/*WS*_2_ flower leaves. Moreover, the high concentration of CNTs could lead to the aggregation in the prepared solution. Therefore, the increased quantity of CNTs in the samples led to the size reduction and the absence of nanoflowers in the frame network. Moreover, the separate growth of *W*_2_*C*/*WS*_2_ was observed as a result of CNT aggregation, as shown in Figure S1.

**Figure 2:**
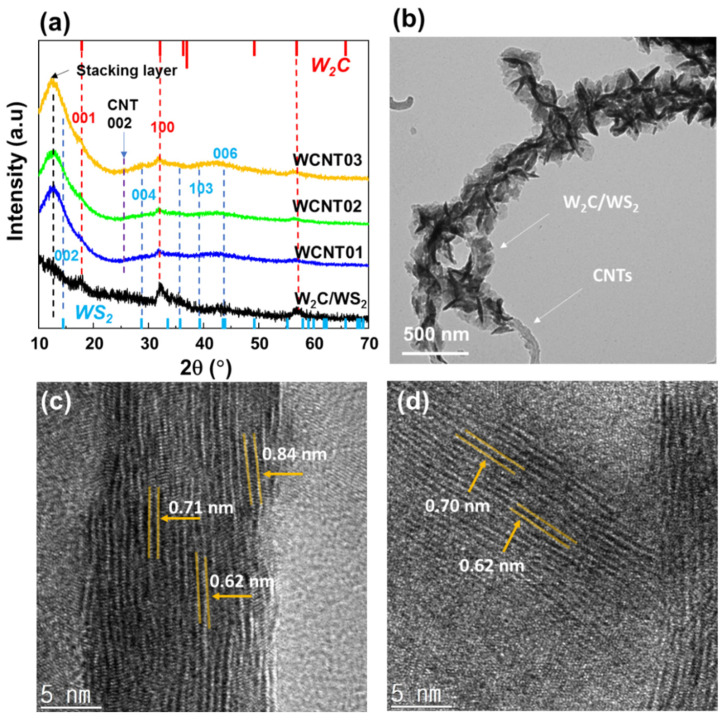
(**a**) XRD patterns of *W*_2_*C*/*WS*_2_ and WCNT01/02/03; (**b**) TEM images and (**c**,**d**) high-resolution TEM (HR-TEM) images of WCNT01.

Figure 2a shows the XRD patterns and TEM/HR-TEM images of the *W*_2_*C*/*WS*_2_ alloy flowers and WCNT samples. The XRD patterns of the *W*_2_*C*/*WS*_2_ alloys were confirmed by the standard *W*_2_*C* and *WS*_2_ peaks, as reported in previous studies [28,42,43]. The (001) and (100) peaks of *W*_2_*C* are clearly observed. The (002) peak of *WS*_2_ overlapped with the stacking layer peak at ~12.6°, whereas the (004), (103), and (006) planes were clearly observed. The (002) peak of the CNTs was not clear until 15% CNT content was used in the samples. The WCNT03 sample showed a low-intensity peak at that position. Furthermore, the high crystallinity of *W*_2_*C* and *WS*_2_ and their large sizes also contributed to the high peak intensity, leading to decreased CNT peaks. In addition, the samples with CNTs showed significantly improved peaks for the stacking layer at 2θ = 12.6°. According to Bragg’s law, *d =* λ/2*sin* θ (where *d* is the lattice spacing, λ is the incident X-ray wavelength, and θ is the diffraction angle); the average lattice spacing was ~7.2 Å. This lattice spacing is large compared to the ionic radius of *Li*^+^, which is 0.76 Å, therefore, this spacing could provide a facile path for lithium ions to easily insert/desert into the material structures. The TEM images in Figure 2b–d also confirm the formation of *W*_2_*C*/*WS*_2_ on CNTs network, with the lattice spacing of the stacking layer in the range of 0.62–0.84 nm, which is consistent with the XRD results. Therefore, the presence of CNTs not only created a frame network but also facilitated the growth of *W*_2_*C*/*WS*_2_ alloys, forming average lattice spacing of ~7.2 Å, which is promising for metal-ion-storage applications. The TEM images with energy dispersive x-ray elemental mapping also confirmed the presence of W, S, C atoms on the *W*_2_*C*/*WS*_2_ and CNT structure in Figure S2. The TGA curves of bare *W*_2_*C*/*WS*_2_ and WCNT01 samples were presented in Figure S3. The mass of bare *W*_2_*C*/*WS*_2_ and WCNT01 sample reduced to ~90% and ~80% after the measurement. It is noted that both *W*_2_*C*/*WS*_2_ and CNTs were oxidized during the measurement. Therefore, the different mass percentage after the measurement is proportional to the mass change from CNTs. The amounts of *W*_2_*C*/*WS*_2_ and CNTs in WCNT01 were calculated to be 90 and 10 wt%, respectively. The increased quantity of CNTs could reduce the nanoflowers’ size, leading to an increase in surface area and an improvement in the electrochemical performance of anode materials.

**Figure 3:**
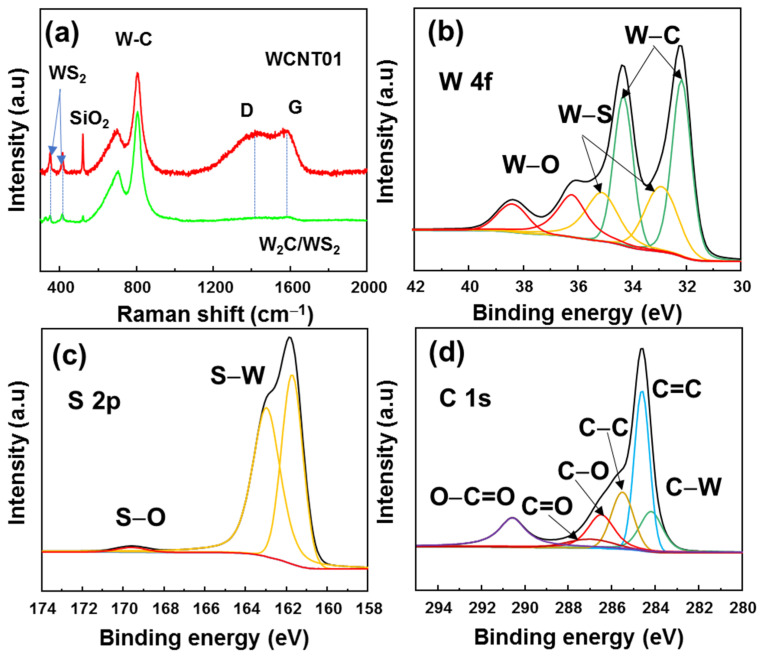
(**a**) Raman spectra of bare *W*_2_*C*/*WS*_2_ and WCNT01 samples. High-resolution XPS spectra of (**b**) W 4f, (**c**) S 2p, and (**d**) C 1s of WCNT01 sample.

To further confirm the structure of *W*_2_*C*/*WS*_2_ on CNTs, Figure 3a shows the Raman spectra of *W*_2_*C*/*WS*_2_ nanoflowers and WCNT01 samples. The optical phonon modes (E12g and A1g) of *WS*_2_ were well-recorded at ~350 and 415 cm^−1^ [44]. The tungsten-carbide vibration modes were also detected at ~700 and ~800 cm^−1^ [10]. The *W*_2_*C*/*WS*_2_ alloys showed a low intensity of carbon sp3 and sp2 peaks, corresponding to the D and G band, respectively. These peaks are highly increased in the WCNT01 sample, indicating the presence of the CNT structure [45,46]. The XPS spectra of WCNT01 material are shown in Figure 3b–d. The W^4+^ peaks can be deconvoluted to the W–C binding and W–S binding, corresponding to the doublets with W 4f_7/2_ at 32.2 and at 33.0 eV, respectively. Moreover, small W^6+^ peaks were observed, which could be due to the oxidation on the surface during sample preparation. Sulfur atoms show a doublet of S 2p_3/2_ and 2p_1/2_ peaks at 161.7 and 163.0 eV, respectively, indicating the S–W binding. A small peak at 169.5 eV was observed due to the surface oxidation of the material. The C 1s spectrum could be deconvoluted into six peaks at 284.1, 284.6, 285.5, 286.5, 287.2, and 290.6 eV, which correspond to the C–W, C=C, C–C, C–O, C=O, and O−C=O binding, respectively. These results are consistent with binding energy of CNT and carbide compounds, indicating the formation of *W*_2_*C*/*WS*_2_ on CNTs [30,45,47].

**Figure 4:**
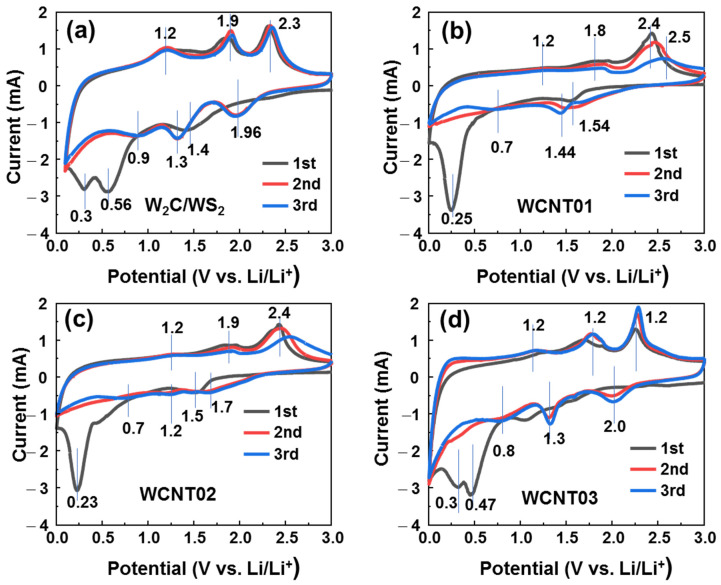
Cyclic voltammograms of the (**a**) *W*_2_*C*/*WS*_2_, (**b**) WCNT01, (**c**) WCNT02, and (**d**) WCNT03 anodes.

To reveal the effectiveness of CNTs in *W*_2_*C*/*WS*_2_ materials, CV of bare and CNT-frame-networked samples was performed (Figure 4). The electrochemical process can be summarized in the following equations:

(1) $$WS_{2}+xLi^{+}+xe^{-}\to Li_{x}WS_{2}$$

(2) $$W_{2}C+yLi^{+}+ye^{-}\to Li_{y}W_{2}C$$

(3) $$Li^{+}+e^{-}+electrolyte\to SEI$$

(4) $$Li_{x}WS_{2}+\left(4-x\right)Li^{+}+\left(4-x\right)e^{-}\to 2Li_{2}S+W$$

(5) $$2Li_{2}S\to 4Li^{+}+4e^{-}+2S$$

In the first cycle of the bare *W*_2_*C/WS*_2_ anode, the cathodic scan showed the insertion of lithium ions into the layered structure of *W*_2_*C* and *WS*_2_ at ~1.5 V (Equations (1) and (2)). The solid-electrolyte-interface (SEI) layer formed at ~0.6 V (Equation (3)) [48,49]. The peak at ~0.3 V is related to the deep insertion of *Li^+^* ions described by Equation (4) [50]. Meanwhile, from the second cycle onward, the cathodic scan demonstrated three major peaks at ~2.0, 1.3, and 0.9 V. As per the previous report, the dissolution of the S atom generated a gel-like SEI layer, which led to the shift of the cathodic peak to ~2.0 V [24]. The peaks at 1.3 and 0.9 V are related to the insertion of *Li* ions into *WS*_2_ and *W*_2_*C* [33]. In the anodic scan, *Li*_2_*S* decomposes at ~ 2.3 V, as shown in Equation (5), and the oxidation of *W* to *W*^4+^ occurs at 1.9 V [23]. The anodic peak at 1.2 V may correspond to the desertion of *Li*^+^ ions from *Li_y_W*_2_*C* as the reverse reaction of Equation (2). The presence of the CNT network increased conductivity and prevented coverage of the gel-like SEI layer. Therefore, the WCNT01 and WCNT02 samples showed cathodic peaks of ~1.6 V and 0.25 V, which are lower than 2.0 V and 0.3 V, as demonstrated by the bare *W*_2_*C/WS*_2_ anode. The decomposition peak of *Li*_2_*S* also shifted to ~2.5 V. Interestingly, the intensity of SEI formation peaks of the WCNT01/02 anodes at ~0.5–0.7 V dramatically decreased in comparison to that of the bare anode. At a CNT content above 15%, the WCNT03 anode showed a similar behavior to the bare *W*_2_*C/WS*_2_ anode, in which the cathodic peak at 2.0 V (Equation (1)) and the peak at ~0.5 V for SEI-layer formation (Equation (3)) appeared again with high intensity. This is attributed to the nonuniform *W*_2_*C/WS*_2_ on the CNTs, which originated from the aggregation of CNTs at high concentrations in the prepared mixture before the hydrothermal process. Thus, a low quantity of CNTs (below 10%) could enhance the electrochemical performance by preventing the coverage of the gel-like SEI layer.

**Figure 5:**
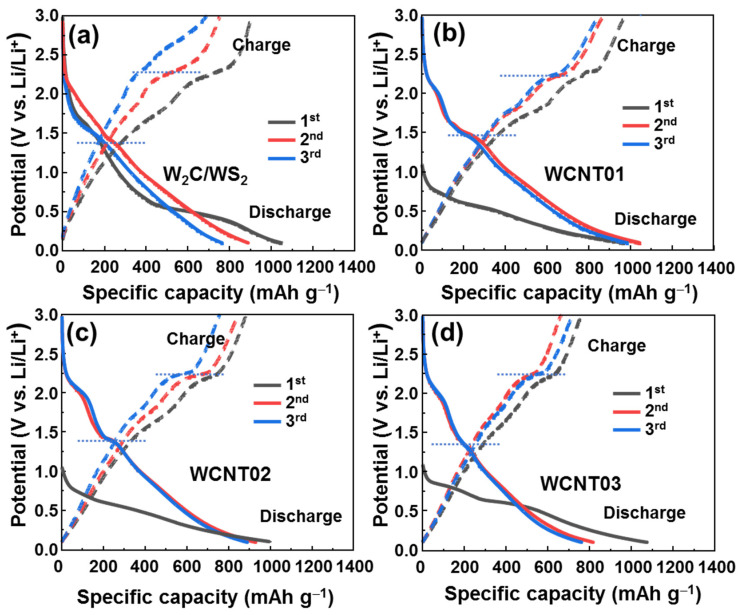
Initial voltage profiles of the (**a**) *W*_2_*C*/*WS*_2_ alloys, (**b**) WCNT01, (**c**) WCNT02, and (**d**) WCNT03 anodes.

The initial voltage profiles of the as-prepared anodes are shown in Figure 5. The WCNT01/02/03 samples showed a low open potential of ~1.1 V in comparison to the *W*_2_*C*/*WS*_2_ sample, which could be attributed to the contact of *W*_2_*C*/*WS*_2_ with CNTs. This behavior was also observed in MoS_2_ and *WS*_2_ grown with graphene or CNTs, as discussed in several reports [45,51,52,53]. The initial discharge capacity of the *W*_2_*C*/*WS*_2_ sample was in the range of 1000–1100 mAh g^−1^. The voltage profiles of the bare *W*_2_*C*/*WS*_2_ alloy anode showed the discharge plateau at ~1.4 V and charge plateau at ~2.3 V. However, these plateaus gradually decreased after the first cycle. In contrast, the plateaus of the WCNT01/02/03 anodes were much more stable, demonstrating a similar flatform during the first three cycles. This indicates that the CNT network optimized the electrochemical reaction, resulting in a stable flatform of the voltage profiles.

**Figure 6:**
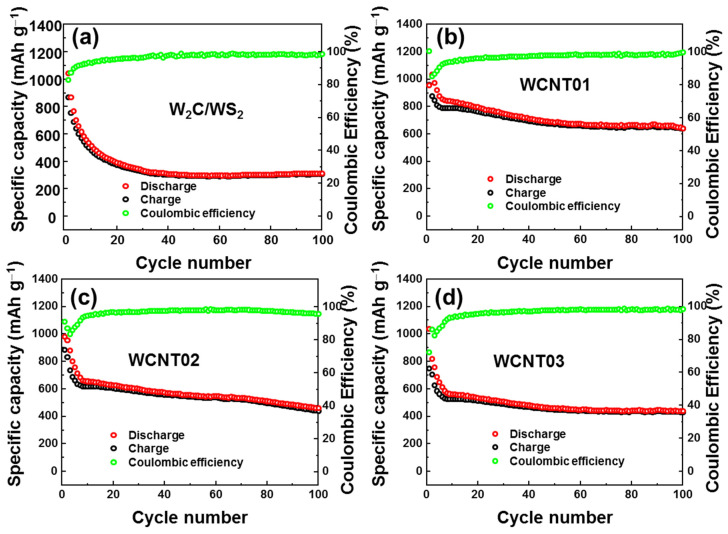
Cyclic performance of the (**a**) *W*_2_*C*/*WS*_2_ alloys, (**b**) WCNT01, (**c**) WCNT02, and (**d**) WCNT03 anodes under the current rate of 0.1 A g^−1^.

The long-term cyclic stability of these four anodes was further evaluated, as illustrated in Figure 6a–d. The bare *W*_2_*C*/*WS*_2_ anode exhibited fast degradation for the first 20 cycles, and only approximately 40% of the initial capacity remained (~400 mAh g^−1^); then, it gradually degraded to ~28% of the initial capacity (~300 mAh g^−1^) after 100 cycles, as shown in Figure 6a. Meanwhile, the WCNT01 electrode underwent a fast degradation in only the first five cycles, and the capacity then slowly degraded to 650 mAh g^−1^ (67% of the initial capacity) after 100 cycles. Both the WCNT02 and WCNT03 anodes showed a fast reduction in capacity for the first ten cycles, followed by a slow reduction to 420 and 410 mAh g^−1^, respectively. These results indicate that a high concentration of CNTs is not necessary and even reduces the overall capacity owing to the lower contribution of the lithium-ion host. Therefore, it was confirmed that only 5% CNTs in the alloys were sufficient to connect the network of *W*_2_*C*/*WS*_2_, prevent fast degradation, and stabilize the capacity. The rate performances of bare *W*_2_*C*/*WS*_2_ and WCNT01 anodes are shown in Figure S4. The bare *W*_2_*C*/*WS*_2_ anode shows a low performance at 1.0 A, which delivered a low capacity ~ 110 mAh g^−1^ and low recovered capacity ~79% when reducing the current rate from 1.0 to 0.1 A g^−1^. on the other hand, the WCNT01 anode with CNT networks could remain at a capacity of ~250 mAh g^−1^ at 1.0 A g^−1^ and recovered ~92% capacity when reducing the current rate to 0.1 A. Moreover, the WCNT01 anode shows a trend to recover 100% capacity when increasing the number of cycles at 0.1 A g^−1^. Furthermore, the capacity of the composite anode continuously degrades with the increase in cycles. This could be due to the main two reasons: First, the lithium-counter electrode could be degraded due to the imperfect recovery of *Li* and the SEI-layer formation on the surface [54,55,56]. Second, it could be due to the degradation of active materials. The CNT network can prevent the formation of a gel-like polymeric layer. However, the sulfur atom could be slowly dissolved into electrolyte during cycling, as discussed for bare *WS*_2_ materials. Due to the conversion type of anode materials, the *WS*_2_ will be converted to W-*Li* alloys and *Li*_2_*S* when inserting the *Li* ions. Therefore, it is believed that the cycling process could slightly change the morphologies and material types. However, with the stability of CNT networks, it can prevent these changes with a slow rate.

**Figure 7:**
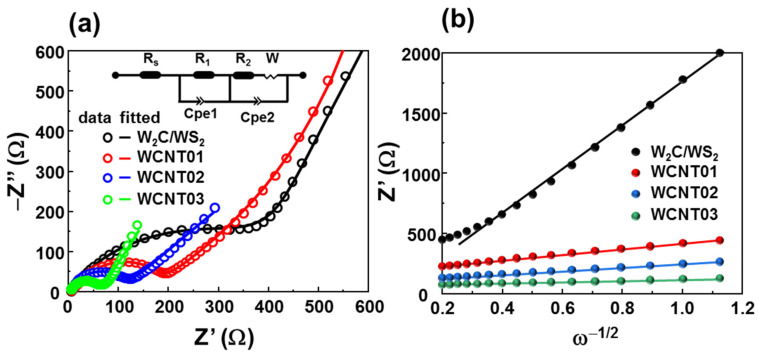
(**a**) Nyquist plots and (**b**) *Z*′ vs. $\omega^{-1/2}$ plots of the bare *W*_2_*C*/*WS*_2_ alloys and WCNT01/02/03 anodes.

Impedance measurements further confirmed the change in the electrical properties of the CNT networks in the *W*_2_*C*/*WS*_2_ materials, as shown in Figure 7a. The equivalent circuit was used with a modified Randle’s model, which contains a series resistance *R_s_*, charge-transfer resistance *R*_1_, and SEI-layer resistance *R*_2_ with a Warburg diffusion element and constant-phase elements Cpe1 and Cpe2. The extracted charge-transfer resistances of the bare *W*_2_*C*/*WS*_2_ and WCNT01/02/03 were 441.3, 125.9, 106.3, and 60.8 Ω, respectively. The enhancement in lithium diffusion can be estimated using the following equation [57,58,59]:

(6) $$D=\frac{R^{2}T^{2}}{2A^{2}n^{4}F^{4}C^{2}\sigma^{2}}$$

where *R* and *F* are the gas constant and Faraday constant, respectively, *T* is the absolute temperature, *A* is the effective area of the working electrode, *n* is the electronic transport ratio during the redox process, *C* is the molar density of *Li^+^* in the electrode, and σ is the Warburg factor associated with the impedance of the cell, which can be obtained from the following equation [60]:

(7) $$Z^{\prime }=R_{s}+R_{1}+R_{2}+\sigma \omega^{-1/2}$$

Figure 7b shows the fitting line of *Z*′ vs. $\omega^{-1/2}$, in which the slopes of the lines of the bare *W*_2_*C*/*WS*_2_ and WCNT01/02/03 anodes are 1697.1, 389.9, 246.5, and 239.6, respectively. According to Equation (6), the diffusion coefficients of the lithium ions in WCNT01/02/03 are proportional to $\sigma^{-1/2}$ and were approximately 19, 49, and 50 times higher than those of the bare *W*_2_*C*/*WS*_2_ anode, respectively. Even though the WCNT02/03 samples showed a great improvement in the lithium diffusion coefficient, their stability in terms of structure and electrochemical properties was not as suitable as that of the WCNT01 electrode. Therefore, WCNT01 is recommended as the best combination of CNTs with *W*_2_*C*/*WS*_2_ alloys for high-performance anodes in lithium-ion cells.

In order to investigate the lithium-storage mechanism, the CV curves at scan rates from 10 to 100 mV s^−1^, and the logarithm plot of peak currents with logarithm of scan rates are shown in Figure S5. The capacitive and diffusion contribution can be evaluated by the b factor in the following equation [61]:

(8) $$i=kv^{b}$$

where *i* is the current density, *v* is the scan rate, *k* and *b* are adjustable factors. When *b* = 1, the storage mechanism is capacitive; when *b* = 0.5, the storage mechanism is diffusion. By the logarithm of Equation (8), the *b* factor can be obtained by plotting the fitting line of log(*i*) vs. log(*v*). The *b* values are 0.9 and 0.52 with cathodic peak and anodic peak, respectively. In the cathodic process, the cell behavior can be considered as a capacitor, while in the anodic process, the diffusion-controlled process is major contributor to the current. Therefore, it is noted that the high reversible capacity of the WCNT01 anode was based on capacitive behavior.

The comparison of the research on *WS*_2_-based materials for the LIB anode is shown in Table 1. It clearly illustrates that the bare 2D or oxygen-modified *WS*_2_ have low electrochemical performances, which reveal their storage capability only for 20 cycles. The optimized compositions of *WS*_2_ with other stable materials are required to enhance their cyclability and rate performance. Most compositions of *WS*_2_ with graphene, carbon, or CNTs can form with some modifications such as N-doping or three-dimensional morphologies, where stable capacity can go up to ~960 mAh g^−1^. In our study, the ternary compound with *W*_2_*C*, *WS*_2_, and CNTs for the LIB anode is not the best material but shows a comparative result. Moreover, the simple preparation method could be an advantage for the application in lithium storage. Therefore, it is noted that control of the flowers’ sizes and/or the compositions of functionalized CNTs and *W*_2_*C*/*WS*_2_ could be effective ways to further enhance their electrochemical performances for LIBs.

## 4. Conclusions

In this study, *W*_2_*C*/*WS*_2_ was synthesized in situ using CNT networks via a hydrothermal method. The presence of CNTs led to a decrease in the number of multi-edge nanoflowers with a size range of 200–400 nm. The CNT networks enhanced the conductivity of anode materials, which in turn reduced the cathodic peak intensity from 2.0 to ~1.6 V. The impedance spectra also suggest that the lithium-ion diffusion in the WCNT01/02/03 samples was 19, 49, and 50 times higher than that of the *W*_2_*C*/*WS*_2_ sample, respectively. WCNT01 anodes with 5% CNTs showed the best performance, with a capacity of 650 mAh g^−1^ (67% of the initial value) remaining after 100 cycles. These results suggest that the utilization of CNT networks and a simple hydrothermal method can be appropriate for improving the overall stability of metal-sulfide anode materials.

## Figures and Tables

**Table 1:** Comparison of electrochemical performance of *WS*_2_-based composite materials for lithium-ion batteries.

Anode Materials	Current Density (mA g^−1^)	Initial Discharge Capacity (mAh g^−1^)	Cycle Number	Specific Capacity (mAh g^−1^)	References
*WS*_2_ nanoflakes	47.5	~1700	20	~700	[22]
Oxygen-functionalized *WS*_2_	50	~920	20	~220	[62]
Hierarchical *WS*_2_ on 3D graphene	100	~800	100	~740	[63]
Mesoporous *WS*_2_	100	~1300	100	~800	[23]
N-graphene/*WS*_2_	100	~1300	100	~800	[64]
N-carbon sphere/*WS*_2_	100	~735	100	~630	[65]
N-graphene/*WS*_2_	100	~950	100	~960	[66]
N-carbon/*WS*_2_	100	~1000	100	~640	[67]
*W*_2_*C*/*WS*_2_/CNTs	100	~1000	100	~650	This work

## Data Availability

The data presented in this study are available on request from the corresponding author.

## Supplementary Materials

The following supporting information can be downloaded at: https://www.mdpi.com/article/10.3390/nano12061003/s1, Figure S1: SEM image of WCNT03 sample with and without CNT area; Figure S2: (a) TEM image, (b) Scanning TEM image and elemental mapped images (c) W-L, (d) S-K and (e) C-K of *W*_2_*C*/*WS*_2_ nanolowers on CNTs; Figure S3: TGA analysis of the synthesized *W*_2_*C*/*WS*_2_ and WCNT01 materials; Figure S4: Rate performance of (a) bare *W*_2_*C*/*WS*_2_ and (b)WCNT01 anodes at different current rate from 0.1 to 1.0 A g^−1^; Figure S5: (a) CV curves of WCNT01 anode at different scan rate from 20–100 mV s^−1^ and (b) plots of log(current) with log(scan-rate).

## References

[1] Kuc A., Heine T. (2015). The electronic structure calculations of two-dimensional transition-metal dichalcogenides in the presence of external electric and magnetic fields. Chem. Soc. Rev..

[2] Haque F., Daeneke T., Kalantar-zadeh K., Ou J.Z. (2017). Two-Dimensional Transition Metal Oxide and Chalcogenide-Based Photocatalysts. Nano-Micro Lett..

[3] Wang Q.H., Kalantar-Zadeh K., Kis A., Coleman J.N., Strano M.S. (2012). Electronics and optoelectronics of two-dimensional transition metal dichalcogenides. Nat. Nanotechnol..

[4] Chhowalla M., Shin H.S., Eda G., Li L.J., Loh K.P., Zhang H. (2013). The chemistry of two-dimensional layered transition metal dichalcogenide nanosheets. Nat. Chem..

[5] Quesne M.G., Roldan A., de Leeuw N.H., Catlow C.R.A. (2018). Bulk and surface properties of metal carbides: Implications for catalysis. Phys. Chem. Chem. Phys..

[6] Yu Z., Xinhui X., Fan S., Jiye Z., Jiangping T., Jin F.H. (2016). Transition Metal Carbides and Nitrides in Energy Storage and Conversion. Adv. Sci..

[7] Gao G., O’Mullane A.P., Du A. (2017). 2D MXenes: A New Family of Promising Catalysts for the Hydrogen Evolution Reaction. ACS Catal..

[8] Pan H. (2016). Ultra-high electrochemical catalytic activity of MXenes. Sci. Rep..

[9] Nguyen T.P., Tuan Nguyen D.M., Tran D.L., Le H.K., Vo D.-V.N., Lam S.S., Varma R.S., Shokouhimehr M., Nguyen C.C., Le Q.V. (2020). MXenes: Applications in electrocatalytic, photocatalytic hydrogen evolution reaction and CO_2_ reduction. Mol. Catal..

[10] Chen Z., Gong W., Cong S., Wang Z., Song G., Pan T., Tang X., Chen J., Lu W., Zhao Z. (2020). Eutectoid-structured WC/W_2_C heterostructures: A new platform for long-term alkaline hydrogen evolution reaction at low overpotentials. Nano Energy.

[11] Hussain S., Shaikh S.F., Vikraman D., Mane R.S., Joo O.-S., Naushad M., Jung J. (2015). Sputtering and sulfurization-combined synthesis of a transparent WS_2_ counter electrode and its application to dye-sensitized solar cells. RSC Adv..

[12] Gowtham B., Balasubramani V., Ramanathan S., Ubaidullah M., Shaikh S.F., Sreedevi G. (2021). Dielectric relaxation, electrical conductivity measurements, electric modulus and impedance analysis of WO_3_ nanostructures. J. Alloys Compd..

[13] Al-Tahan M.A., Dong Y., Shrshr A.E., Liu X., Zhang R., Guan H., Kang X., Wei R., Zhang J. (2022). Enormous-sulfur-content cathode and excellent electrochemical performance of Li-S battery accouched by surface engineering of Ni-doped WS^2^@rGO nanohybrid as a modified separator. J. Colloid Interface Sci..

[14] Bai Y., Liu C., Chen T., Li W., Zheng S., Pi Y., Luo Y., Pang H. (2021). MXene-Copper/Cobalt Hybrids via Lewis Acidic Molten Salts Etching for High Performance Symmetric Supercapacitors. Angew. Chem. Int. Ed..

[15] Li X., Yang X., Xue H., Pang H., Xu Q. (2020). Metal–organic frameworks as a platform for clean energy applications. EnergyChem.

[16] Chen J., Chen Y., Feng L.-W., Gu C., Li G., Su N., Wang G., Swick S.M., Huang W., Guo X. (2020). Hole (donor) and electron (acceptor) transporting organic semiconductors for bulk-heterojunction solar cells. EnergyChem.

[17] Kim S., Park J., Hwang J., Lee J. (2021). Effects of functional supports on efficiency and stability of atomically dispersed noble-metal electrocatalysts. EnergyChem.

[18] Zheng S., Sun Y., Xue H., Braunstein P., Huang W., Pang H. (2021). Dual-ligand and hard-soft-acid-base strategies to optimize metal-organic framework nanocrystals for stable electrochemical cycling performance. Natl. Sci. Rev..

[19] Kim H., Choi W., Yoon J., Um J.H., Lee W., Kim J., Cabana J., Yoon W.S. (2020). Exploring Anomalous Charge Storage in Anode Materials for Next-Generation Li Rechargeable Batteries. Chem. Rev..

[20] Zheng Y., Li Y., Yao J., Huang Y., Xiao S. (2018). Facile synthesis of porous tubular NiO with considerable pseudocapacitance as high capacity and long life anode for lithium-ion batteries. Ceram. Int..

[21] Nguyen T.P., Giang T.T., Kim I.T. (2022). Restructuring NiO to LiNiO_2_: Ultrastable and reversible anodes for lithium-ion batteries. Chem. Eng. J..

[22] Feng C., Huang L., Guo Z., Liu H. (2007). Synthesis of tungsten disulfide (WS_2_) nanoflakes for lithium-ion battery application. Electrochem. Commun..

[23] Liu H., Su D., Wang G., Qiao S.Z. (2012). An ordered mesoporous WS_2_ anode material with superior electrochemical performance for lithium-ion batteries. J. Mater. Chem..

[24] George C., Morris A.J., Modarres M.H., De Volder M. (2016). Structural Evolution of Electrochemically Lithiated MoS_2_ Nanosheets and the Role of Carbon Additive in Li-Ion Batteries. Chem. Mater..

[25] Jia L., Liu B., Zhao Y., Chen W., Mou D., Fu J., Wang Y., Xin W., Zhao L. (2020). Structure design of MoS_2_@Mo_2_C on nitrogen-doped carbon for enhanced alkaline hydrogen evolution reaction. J. Mater. Sci..

[26] Liu J., Wang P., Gao L., Wang X., Yu H. (2022). In situ sulfuration synthesis of heterostructure MoS_2_–Mo_2_C@C for boosting the photocatalytic H_2_ production activity of TiO_2_. J. Mater. Chem. C.

[27] Ihsan M., Wang H., Majid S.R., Yang J., Kennedy S.J., Guo Z., Liu H.K. (2016). MoO_2_/Mo_2_C/C spheres as anode materials for lithium-ion batteries. Carbon.

[28] Li Y., Wu X., Zhang H., Zhang J. (2018). Interface Designing over WS_2_/W_2_C for Enhanced Hydrogen Evolution Catalysis. ACS Appl. Energy Mater..

[29] Zhao Z., Qin F., Kasiraju S., Xie L., Alam M.K., Chen S., Wang D., Ren Z., Wang Z., Grabow L.C. (2017). Vertically Aligned MoS_2_/Mo_2_C hybrid Nanosheets Grown on Carbon Paper for Efficient Electrocatalytic Hydrogen Evolution. ACS Catal..

[30] Cheng Y., Pang K., Wu X., Zhang Z., Xu X., Ren J., Huang W., Song R. (2018). In Situ Hydrothermal Synthesis MoS_2_/Guar Gum Carbon Nanoflowers as Advanced Electrocatalysts for Electrocatalytic Hydrogen Evolution. ACS Sustain. Chem. Eng..

[31] Faizan M., Hussain S., Vikraman D., Ali B., Kim H.-S., Jung J., Nam K.-W. (2021). MoS_2_@Mo_2_C hybrid nanostructures formation as an efficient anode material for lithium-ion batteries. J. Mater. Res. Technol..

[32] Nguyen T.P., Choi K.S., Kim S.Y., Lee T.H., Jang H.W., Van Le Q., Kim I.T. (2020). Strategy for controlling the morphology and work function of W_2_C/WS_2_ nanoflowers. J. Alloys Compd..

[33] Nguyen T.P., Kim I.T. (2020). W_2_C/WS_2_ Alloy Nanoflowers as Anode Materials for Lithium-Ion Storage. Nanomaterials.

[34] Sun D., Wang M., Li Z., Fan G., Fan L.-Z., Zhou A. (2014). Two-dimensional Ti_3_C_2_ as anode material for Li-ion batteries. Electrochem. Commun..

[35] Wang Y., Li Y., Qiu Z., Wu X., Zhou P., Zhou T., Zhao J., Miao Z., Zhou J., Zhuo S. (2018). Fe_3_O_4_@Ti_3_C_2_ MXene hybrids with ultrahigh volumetric capacity as an anode material for lithium-ion batteries. J. Mater. Chem. A.

[36] Kong F., He X., Liu Q., Qi X., Sun D., Zheng Y., Wang R., Bai Y. (2018). Enhanced reversible Li-ion storage in Si@Ti_3_C_2_ MXene nanocomposite. Electrochem. Commun..

[37] Zhang Y. (2017). First principles prediction of two-dimensional tungsten carbide (W_2_C) monolayer and its Li storage capability. Comput. Condens. Matter.

[38] Bai Y.-L., Liu Y.-S., Ma C., Wang K.-X., Chen J.-S. (2018). Neuron-Inspired Design of High-Performance Electrode Materials for Sodium-Ion Batteries. ACS Nano.

[39] Landi B.J., Ganter M.J., Cress C.D., Di Leo R.A., Raffaelle R.P. (2009). Carbon nanotubes for lithium-ion batteries. Energy Environ. Sci..

[40] Lu C., Liu W.-W., Li H., Tay B.K. (2014). A binder-free CNT network–MoS_2_ composite as a high performance anode material in lithium ion batteries. Chem. Commun..

[41] Chen Y., Hu X., Evanko B., Sun X., Li X., Hou T., Cai S., Zheng C., Hu W., Stucky G.D. (2018). High-rate FeS_2_/CNT neural network nanostructure composite anodes for stable, high-capacity sodium-ion batteries. Nano Energy.

[42] Yan G., Wu C., Tan H., Feng X., Yan L., Zang H., Li Y. (2017). N-Carbon coated P-W_2_C composite as efficient electrocatalyst for hydrogen evolution reactions over the whole pH range. J. Mater. Chem. A.

[43] Nguyen T.P., Kim S.Y., Lee T.H., Jang H.W., Le Q.V., Kim I.T. (2020). Facile synthesis of W_2_C@WS_2_ alloy nanoflowers and their hydrogen generation performance. Appl. Surf. Sci..

[44] Nguyen T.P., Sohn W., Oh J.H., Jang H.W., Kim S.Y. (2016). Size-Dependent Properties of Two-Dimensional MoS_2_ and WS_2_. J. Phys. Chem. C.

[45] Ren J., Ren R.-P., Lv Y.-K. (2019). WS_2_-decorated graphene foam@CNTs hybrid anode for enhanced lithium-ion storage. J. Alloys Compd..

[46] Vaziri H.S., Shokuhfar A., Afghahi S.S.S. (2020). Synthesis of WS_2_/CNT hybrid nanoparticles for fabrication of hybrid aluminum matrix nanocomposite. Mater. Res. Express.

[47] Zhang L.-N., Ma Y.-Y., Lang Z.-L., Wang Y.-H., Khan S.U., Yan G., Tan H.-Q., Zang H.-Y., Li Y.-G. (2018). Ultrafine cable-like WC/W_2_C heterojunction nanowires covered by graphitic carbon towards highly efficient electrocatalytic hydrogen evolution. J. Mater. Chem. A.

[48] Chen Y., Song B., Tang X., Lu L., Xue J. (2014). Ultrasmall Fe_3_O_4_ Nanoparticle/MoS_2_ Nanosheet Composites with Superior Performances for Lithium-Ion Batteries. Small.

[49] Jin Y., Li S., Kushima A., Zheng X., Sun Y., Xie J., Sun J., Xue W., Zhou G., Wu J. (2017). Self-healing SEI enables full-cell cycling of a silicon-majority anode with a coulombic efficiency exceeding 99.9%. Energy Environ. Sci..

[50] Stephenson T., Li Z., Olsen B., Mitlin D. (2014). Lithium-ion battery applications of molybdenum disulfide (MoS_2_) nanocomposites. Energy Environ. Sci..

[51] Xiao J., Wang X.J., Yang X.Q., Xun S.D., Liu G., Koech P.K., Liu J., Lemmon J.P. (2011). Electrochemically Induced High Capacity Displacement Reaction of PEO/MoS_2_/Graphene Nanocomposites with Lithium. Adv. Funct. Mater..

[52] Ren J., Ren R.-P., Lv Y.-K. (2018). A flexible 3D graphene@CNT@MoS_2_ hybrid foam anode for high-performance lithium-ion battery. Chem. Eng. J..

[53] Wang Y., Chen B., Seo D.H., Han Z.J., Wong J.I., Ostrikov K., Zhang H., Yang H.Y. (2016). MoS_2_-coated vertical graphene nanosheet for high-performance rechargeable lithium-ion batteries and hydrogen production. NPG Asia Mater..

[54] Peled E., Menkin S. (2017). Review—SEI: Past, Present and Future. J. Electrochem. Soc..

[55] Lin D., Liu Y., Cui Y. (2017). Reviving the lithium metal anode for high-energy batteries. Nat. Nanotechnol..

[56] Liu J., Bao Z., Cui Y., Dufek E.J., Goodenough J.B., Khalifah P., Li Q., Liaw B.Y., Liu P., Manthiram A. (2019). Pathways for practical high-energy long-cycling lithium metal batteries. Nat. Energy.

[57] Bisquert J., Garcia-Belmonte G., Bueno P., Longo E., Bulhões L.O.S. (1998). Impedance of constant phase element (CPE)—Blocked diffusion in film electrodes. J. Electroanal. Chem..

[58] Piao T., Park S.M., Doh C.H., Moon S.I. (1999). Intercalation of Lithium Ions into Graphite Electrodes Studied by AC Impedance Measurements. J. Electrochem. Soc..

[59] Ye B., Xu L., Wu W., Ye Y., Yang Z., Ai J., Qiu Y., Gong Z., Zhou Y., Huang Q. (2022). Encapsulation of 2D MoS_2_ nanosheets into 1D carbon nanobelts as anodes with enhanced lithium/sodium storage properties. J. Mater. Chem. C.

[60] Rui X.H., Ding N., Liu J., Li C., Chen C.H. (2010). Analysis of the chemical diffusion coefficient of lithium ions in Li_3_V_2_(PO_4_)_3_ cathode material. Electrochim. Acta.

[61] Abdelaal M.M., Hung T.-C., Mohamed S.G., Yang C.-C., Huang H.-P., Hung T.-F. (2021). A Comparative Study of the Influence of Nitrogen Content and Structural Characteristics of NiS/Nitrogen-Doped Carbon Nanocomposites on Capacitive Performances in Alkaline Medium. Nanomaterials.

[62] Bhandavat R., David L., Singh G. (2012). Synthesis of Surface-Functionalized WS_2_ Nanosheets and Performance as Li-Ion Battery Anodes. J. Phys. Chem. Lett..

[63] Huang G., Liu H., Wang S., Yang X., Liu B., Chen H., Xu M. (2015). Hierarchical architecture of WS_2_ nanosheets on graphene frameworks with enhanced electrochemical properties for lithium storage and hydrogen evolution. J. Mater. Chem. A.

[64] Chen D., Ji G., Ding B., Ma Y., Qu B., Chen W., Lee J.Y. (2013). In situ nitrogenated graphene—Few-layer WS_2_ composites for fast and reversible Li^+^ storage. Nanoscale.

[65] Zeng X., Ding Z., Ma C., Wu L., Liu J., Chen L., Ivey D.G., Wei W. (2016). Hierarchical Nanocomposite of Hollow N-Doped Carbon Spheres Decorated with Ultrathin WS_2_ Nanosheets for High-Performance Lithium-Ion Battery Anode. ACS Appl. Mater. Interfaces.

[66] Debela T.T., Lim Y.R., Seo H.W., Kwon I.S., Kwak I.H., Park J., Cho W.I., Kang H.S. (2018). Two-Dimensional WS_2_@Nitrogen-Doped Graphite for High-Performance Lithium-Ion Batteries: Experiments and Molecular Dynamics Simulations. ACS Appl. Mater. Interfaces.

[67] Zhao Z., Wang F., Yuan H., Yang Z., Qin Y., Zheng X., Yang Y. (2021). N-Doped Carbon—WS_2_ Nanosheet Composites for Lithium-Ion Storage. ACS Appl. Nano Mater..

