# Supplementary material for: In Situ Growth of W2C/WS2 with Carbon-Nanotube Networks for Lithium-Ion Storage
Source: Nanomaterials (Basel). 2022 Mar 18;12(6):1003. doi: 10.3390/nano12061003 (PMC8953370; doi:10.3390/nano12061003)
Supplement: Supplementary file 1 [file nanomaterials-12-01003-s001.zip › nanomaterials-1620364-supplementary.pdf]

Supplementary Materials

In Situ Growth of W_2C/WS_2 with Carbon-Nanotube Networks for Lithium-Ion Storage

Thang Phan Nguyen and Il Tae Kim *

Department of Chemical and Biological Engineering, Gachon University, Seongnam-si 13120, Korea; phanthang87@gmail.com
* Correspondence: itkim@gachon.ac.kr

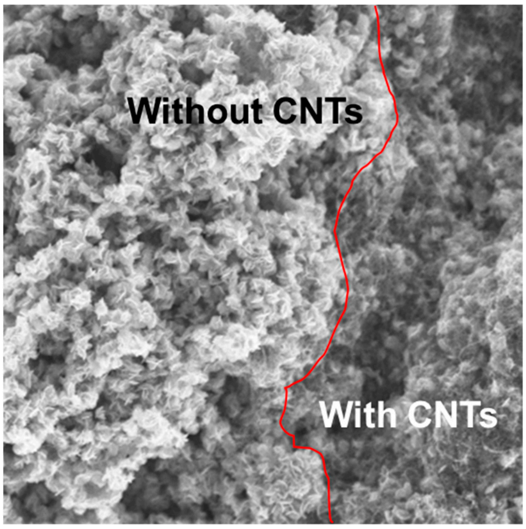

Figure S1. SEM image of WCNT03 sample with and without CNT area.

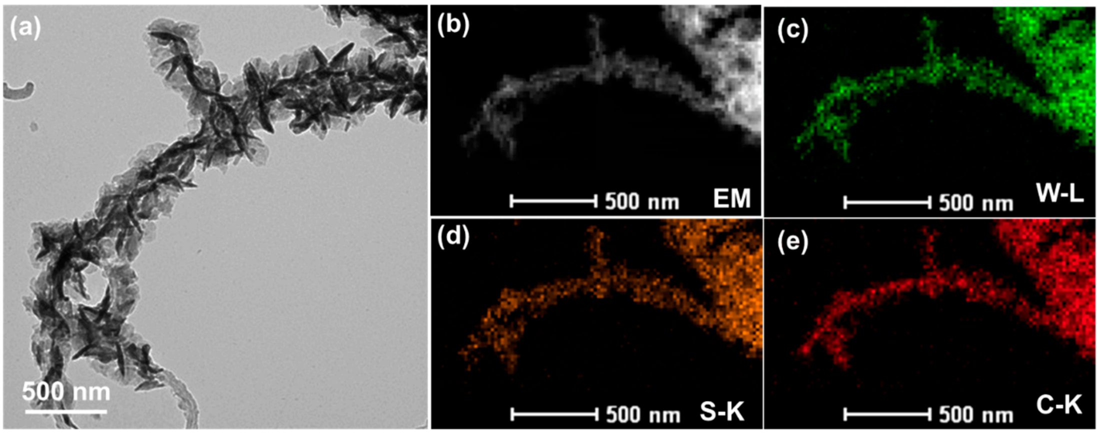

Figure S2. (**a**) TEM image, (**b**) Scanning TEM image and elemental mapped images (**c**) W-L, (**d**) S-K and (**e**) C-K of W_2C/WS_2 nanolowers on CNTs.

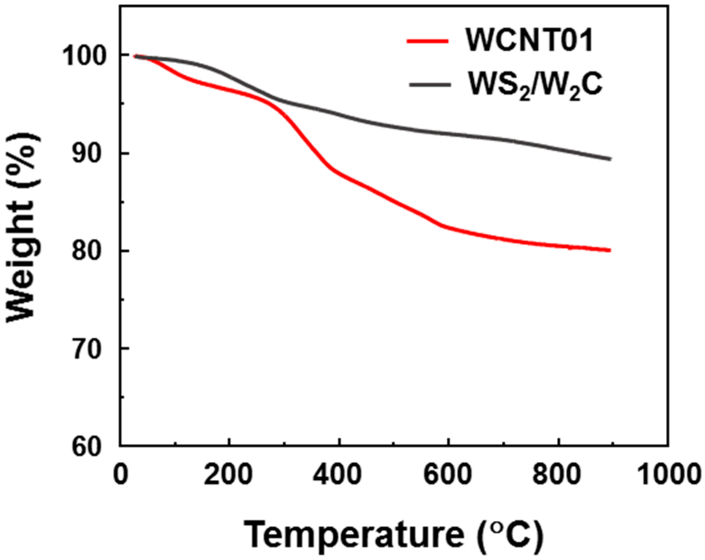

Figure S3. TGA analysis of the synthesized W_2C/WS_2 and WCNT01 materials.

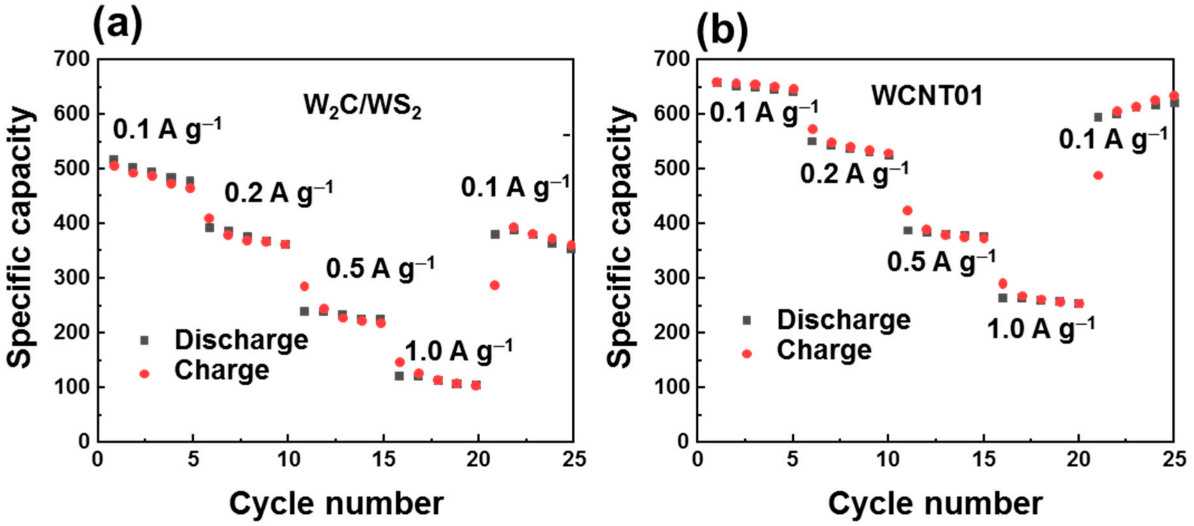

Figure S4. Rate performance of (**a**) bare W_2C/WS_2 and (**b**)WCNT01 anodes at different current rate from 0.1 to 1.0 A g^{-1}.

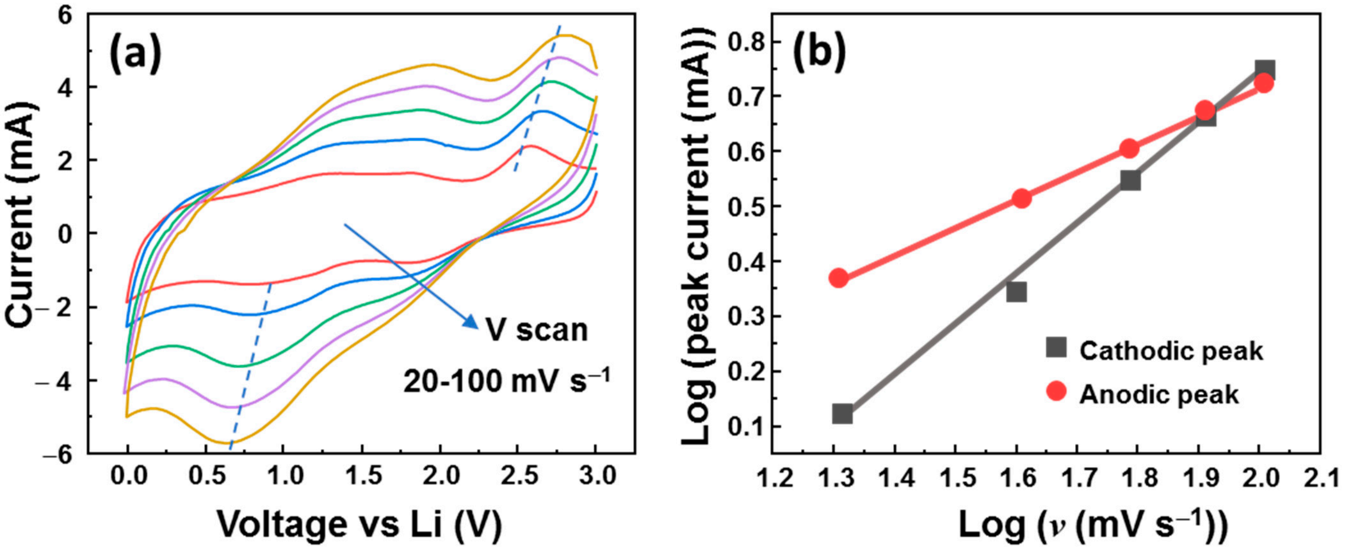

Figure S5. (**a**) CV curves of WCNT01 anode at different scan rate from 20 – 100 mV s^{-1} and (**b**) plots of log(current) with log(scan-rate).
